# Apathy and Related Executive Syndromes in Dementia Associated with Parkinson’s Disease and in Alzheimer’s Disease

**DOI:** 10.3233/BEN-129023

**Published:** 2013

**Authors:** Dario Grossi, Gabriella Santangelo, Anna Maria Barbarulo, Carmine Vitale, Giovanna Castaldo, Maria Grazia Proto, Pietro Siano, Paolo Barone, Luigi Trojano

**Affiliations:** ^1^Department of Psychology, Second University of Naples, CasertaItaly; ^2^Istituto di Diagnosi e Cura “Hermitage Capodimonte”, NaplesItaly; ^3^University “Parthenope”, NaplesItaly; ^4^Neuropsicologia clinica, Azienda Ospedaliera Universitaria OO.RR. S.Giovanni di Dio e Ruggi d’Aragona, SalernoItaly; ^5^Neurodegenerative Center Diseases, University of Salerno, SalernoItaly

**Keywords:** Apathy, Parkinson’s disease, Alzheimer’s disease, executive dysfunctions, frontal lobe

## Abstract

Apathy is defined as a lack of motivation and has been reported to be common in Alzheimer’s disease (AD) and Parkinson's disease (PD). To explore the neuropsychological correlates of apathy in patients with PD related dementia (PDD) and AD and to identify the specific cognitive profile of apathy in the two forms of neurodegenerative disease, 61 non-depressed patients (29 PDD and 32 AD) were selected. Out of these, 29 patients (47.5%) were detected as apathetic (14 PDD-A+ and 15 AD-A+), and 32 patients as non-apathetic (15 PDD-A- and 17 AD-A-). All patients underwent cognitive tasks tapping memory, visuospatial and executive functions, behavioral rating scales and Clinical Judgment for Apathy Syndrome (CJ-AS), an inventory developed to measure severity of apathy.

The four subgroups differed significantly on memory and frontal tasks. The PDD-A+ performed significantly worse than PDD-A- on frontal tasks. The AD-A+ had poorer performance than AD-A- on frontal tasks. Last, PDD-A+ achieved significantly higher scores than AD-A+ on memory tasks. The four groups differed significantly on CJ-AS and behavioral rating scales.

The results showed that apathetic patients with both forms of dementia showed a common neuropsychological and behavioral picture, characterized by defects on frontal tasks, thus strongly supporting the existence of an ‘apathetic syndrome’, characterized by specific cognitive and psychological symptoms.

## 1. Introduction

Apathy has been defined as a lack of motivation leading to a reduction of self-generated voluntary and purposeful behaviors [1,2]. According to Stuss et al. [3], apathetic syndromes can be divided into three subtypes, depending on the domain in which lack of motivation occurs most: 'emotional', 'cognitive' and 'behavioral'. Apathy often manifests after damage to the PreFrontal Cortex (PFC) [3–6] and is considered as a clinical symptom of basal ganglia diseases, such as Huntington's disease, [7–9] and Progressive Supranuclear Palsy (PSP) [10–12]. In Parkinson's disease (PD) apathy is deemed a cardinal non-motor symptom [10, 13–16], occurring since early stages of the disease [17], and has been related to disruption of the PFC-basal ganglia axis [2]. Apathy can be difficult to recognize and to differentiate from depression [18], and for this

ISSN 0953-4180/13/$27.50 © 2013 – IOS Press and the authors. All rights reserved reason its reported prevalence varies from 14% to 70% (mean: 38%) [19]. PD patients with apathy tends to show more severe cognitive symptoms, and particularly executive dysfunction [14,15,20–23], with respect to PD patients without apathy. In a 18-month longitudinal study, Dujardin et al. [21] found apathy to be predictive of development of dementia.

Apathy has also been observed in Alzheimer's disease (AD) since its early stages [24], and is associated with low functional autonomy, severe executive dysfunction and fast cognitive and functional decline [24–27]. Brain imaging studies in AD reported a significant association between apathy and abnormal perfusion in the frontal cortex and in the cingulate area [28–35].

In the present study, we explored the neuropsychological correlates of apathy in patients with PD related dementia (PDD) and AD to the aim of identifying the specific cognitive profile of apathy in the two forms of neurodegenerative disease. Since apathy is classically encompassed among clinical criteria of depression [18], but cognitive and behavioral features of apathy and depression are at least partially divergent [16,23], we selected for the present study only patients without relevant depression, as assessed by standardized questionnaires. Although difficult, differentiating apathy from depression is important, also in view of possible treatment strategies. Depression is thought to be characterized by sadness and negative thoughts about the self, while hallmarks of apathy are considered behavioral lack of initiation and lack of effort, without negative self or event appraisal [36].

The finding of a common neuropsychological and behavioral picture associated with apathy, independently from the kind of neurodegenerative disease, would strongly support the existence of an 'apathetic syndrome', characterized by specific cognitive and psychological symptoms.

## 2. Methods

### 2.1. Patients

Study participants were patients with a diagnosis of either PDD or AD. PDD patients attended the neurology clinic of Department of Neurological Science, University "Federico II", Naples, Italy, whereas AD patients were enrolled at "Azienda Ospedaliera Universitaria OO.RR. S.Giovanni di Dio e Ruggi d'Aragona", Salerno, Italy.

#### 2.1.1. Dementia associated with Parkinson's disease

We screened for the study consecutive outpatients with PDD, diagnosed according to an algorithm recommended by the MDS Task Force [37]. The algorithm comprises five criteria which, if all present, lead to diagnosis of PDD: 1) diagnosis of PD according to standard clinical criteria, such as those set by United Kingdom Parkinson's Disease Society brain bank [38]; 2) development of PD prior to onset of dementia; 3) PD associated with decreased global cognitive efficiency; 4) cognitive deficiency severe enough to impair daily life; 5) presence of impairment in more than one cognitive domain. As a screening for general cognitive impairment we used an Italian standardized version of the Mental State Examination (MMSE), and included in the study PD patients with a total age- and educational-adjusted score $< 23.8$, that is the standard cut-off for the normal range [39].

Patients were excluded if they presented clinically relevant depressive symptomatology, as identified by a score above the cut-off on a standardized questionnaire (see below).

All enrolled patients underwent a neurological examination with the Unified Parkinson's Disease Rating Scale motor subscale (UPDRS-III) [40] to evaluate severity of motor symptoms and were assessed while in the "on" state. Age, level of education, and PD duration were recorded; Levodopa equivalent daily dose (LEDD) was calculated [41].

#### 2.1.2. Alzheimer's disease group

We enrolled patients with diagnosis of probable AD according to NINCDS-ADRDA criteria [42]. To avoid any possible diagnostic bias, and to ensure the highest homogeneity in the patient sample, we excluded from the AD sample all patients with extrapiramidal signs, as in other studies comparing cognitive and behavioral features of PDD and AD [43].

As for the PDD group, patients were excluded if they presented clinically relevant depressive symptomatology, as identified by a score above the cut-off on a standardized questionnaire (see below).

Last, to select two groups of patients with similar general cognitive impairment, for the present study we selected AD patients with an age- and educational-adjusted MMSE score matching that found in PDD group.

### 2.2. Procedures

Several rating scales were administered to patients and caregivers to assess presence of apathy and of other Behavioural and Psychological Symptoms of Dementia (BPSD). All patients also underwent a comprehen-

Table 1
Demographic and clinical characteristics of patients with PD-associated Dementia (PDD) and Alzheimer's disease (AD)

| | PDD ($n = 29$) | AD ($n = 32$) | $U$ test/$\chi^2$ | P |
|---|---|---|---|---|
| Age (yr) | 69.4 ± 8.3 | 69.7 ± 7.1 | 454 | 0.885 |
| Sex (F/M) | 11/18 | 17/15 | 1.414 | 0.234 |
| Education (yr) | 10.4 ± 5.5 | 8.2 ± 4.4 | 373.5 | 0.178 |
| Disease duration (yr) | 7.1 ± 5.2 | – | – | – |
| Hoehn and Yahr score | 1.8 ± 0.6 | – | – | – |
| UPDRS-III score in on phase | 19.4 ± 8.5 | – | – | – |
| (LEDD mg/d) | 599 ± 235.2 | – | – | – |
| MMSE | 22.5 ± 3.9 | 21.6 ± 4.6 | 382 | 0.702 |
| FAB | 9.7 ± 2.7 | 9.2 ± 3.1 | 409 | 0.424 |
| Clock drawing test | 2.6 ± 3 | 3 ± 2.7 | 392 | 0.289 |

UPDRS-III score, Unified Parkinson's Disease Rating Scale; LEDD, Levodopa dose equivalent; MMSE, Mini Mental State Examination; FAB, Frontal Assessment Battery.

sive neuropsychological battery consisting of cognitive tasks for assessment of memory, visuospatial and executive functions.

### 2.2.1. *Neurobehavioral assessment*

Severity of depression was measured by means of the Italian version of Hamilton Depression Rating Scale (HAMD-17) [44]. In reference to the Italian version of HAMD-17, a cut-off value $> 16$ identifies patients with marked depressive symptoms, who were excluded from the study. To ensure that patients with relevant depressive symptoms were excluded from the study, even if patients themselves could not be able to correctly report those symptoms, HAM-D was compiled by the clinician after a semistructured interview with the patient and his/her caregiver.

To identify clinically relevant apathy and to measure its severity we used the Italian Informant and Self-rated versions of the Apathy Evaluation Scale (I-AES and S-AES) [1]. The assessment of 12 neurobehavioral disturbances (delusions, hallucinations, dysphoria, anxiety, agitation/aggression, euphoria, disinhibition, irritability/lability, apathy, and aberrant motor, night time behavior and eating disorders) was performed by means of Neuropsychiatric Inventory, a validated informant-based interview [45]. Functional independence was explored by Instrumental Activity of Daily Living Scale (IADL).

At the end of the assessment, the examiner also recorded whether each patient had showed 3 verbal (i.e., laconic expressions, brief responses to repeated prompts, monotonic prosody) and 3 non-verbal (i.e., poor gestuality, rare spontaneous movements, rare emotional facial expressions) clinical signs of apathy during the interview and neuropsychological assessment. The sum of observed clinical signs (defined as Clinical Judgment of Apathy Syndrome, CJ-AS) was considered as an index of examiner's judgment about severity of apathy (range 0–6).

### 2.2.2. *Neuropsychological assessment*

Frontal lobe functions were assessed by means of Frontal Assessment Battery [46], Semantic [47] and Phonological verbal fluency tasks [48], copying task in the Rey-Osterrieth Complex Figure Test (ROCF) [49], part B of Trail Making Test (TMT) [50], the Stroop Color-Word Test and Inverse Motor Learning Test (IML) [51]. Visuospatial functions were evaluated by means of Raven's Colored Progressive Matrices (RCPM) [48], Apraxia Constructional Test [47]. Memory functions were assessed by means of Corsi's block-tapping test (Corsi's test) [47], Bysillabic Word Test (VS) [47], immediate and delayed recall of 15 Rey' word list [48].

### 2.3. *Statistical analysis*

Differences in the distribution of categorical variables among groups were assessed by means of chi-square. For the analysis of differences among groups regarding demographic and neuropsychological variables we used non-parametric tests (Kruskal-Wallis H test to compare four samples, and the Mann-Whitney $U$ test to compare two samples) to avoid biases due to the small sample size.

## 3. Results

From an initial sample of 109 patients (45 PDD and 64 AD), we identified 61 non-depressed patients (29 PDD and 32 AD); PDD or AD did not differ for demographic, clinical and cognitive features (Table 1). The cut-off scores of both I-AES and S-AES ($> 38$)

Table 2
Demographic and clinical characteristics of patients with and without apathy

| | Patients with apathy ($n = 29$) | Patients without apathy ($n = 32$) | $U$ test | P |
|---|---|---|---|---|
| Age (yr) | 69.8 ± 8.7 | 69.3 ± 6.7 | 424.5 | 0.568 |
| Education (yr) | 8.4 ± 4.9 | 10.1 ± 5.2 | 366.5 | 0.147 |
| MMSE | 21.4 ± 3.9 | 22.8 ± 4.5 | 315 | 0.146 |
| FAB | 9.1 ± 2.9 | 9.8 ± 2.9 | 396 | 0.323 |
| Clock drawing test | 2.4 ± 2.6 | 3.1 ± 2.9 | 393 | 0.296 |
| Corsi's block-tapping test | 3.4 ± 1.4 | 3.7 ± 1.3 | 396.5 | 0.293 |
| Verbal span for bysillabic words | 3.1 ± 1 | 3.2 ± 1 | 403 | 0.339 |
| Immediate recall | 19.1 ± 7.5 | 20.2 ± 8.8 | 428 | 0.603 |
| Delayed recall | 2.7 ± 2.7 | 3 ± 2.6 | 429.5 | 0.612 |
| RCPM | 13 ± 5.3 | 16.6 ± 5.6 | 293.5 | **0.014** |
| Apraxia constructional task | 6.1 ± 3.4 | 7.2 ± 3.6 | 376.5 | 0.204 |
| ROCF-copy task | 11.6 ± 8.4 | 16 ± 11.1 | 374 | 0.193 |
| ROCF-delayed recall | 1.9 ± 2.9 | 3.9 ± 4.8 | 353.5 | 0.093 |
| Stroop test-interference task | 3.5 ± 4.3 | 4.4 ± 4.3 | 390 | 0.281 |
| TMT:A | 168.6 ± 116.8 | 147.7 ± 132.5 | 328 | 0.158 |
| TMT:B | 429.9 ± 141.9 | 338.9 ± 141.9 | 224 | **0.024** |
| TMT:B-A | 228.5 ± 188.3 | 279.3 ± 250.7 | 331.5 | 0.055 |
| Phonological fluency | 12.3 ± 8.2 | 15.3 ± 9.4 | 372 | 0.183 |
| Semantic fluency task | 8.1 ± 3 | 11 ± 7.1 | 326 | **0.046** |
| IML: correct responses | 11 ± 7.2 | 16.7 ± 7.5 | 261 | **0.003** |
| IML: no reversal responses | 13.3 ± 7.6 | 6.6 ± 7.4 | 236.5 | **0.001** |
| IADL | 4.3 ± 1.9 | 4.2 ± 2.4 | 458.5 | 0.936 |

MMSE, Mini Mental State Examination; FAB, Frontal Assessment Battery; RCPM, Raven's Colored Progressive Matrices; IML, Inverse Motor Learning test; ROCF, Rey-Osterrieth complex figure test; TMT, Trail Making Test.

allowed to detect 29 patients (47.5% of the sample) affected by apathy (14 PDD and 15 AD), whereas 32 patients were considered non-apathetic (15 PDD and 17 AD).

Demographic characteristics did not differ in apathetic and non-apathetic patients (Table 2), but neuropsychological findings showed that apathetic patients performed significantly worse than non-apathetic patients on semantic fluency test ($p = 0.046$), RCPM ($p = 0.014$), TMT:B ($p = 0.024$), and on both measures derived from IML test (correct responses: $p = 0.003$; no reversal responses: $p = 0.001$).

To investigate whether the type of neurodegenerative disease (PDD vs. AD) influenced the cognitive profile of apathetic and non-apathetic patients, we compared neuropsychological scores achieved by apathetic and non-apathetic patient divided for their diagnosis in the following four subgroups: 1) PDD apathetic patients (PDD-$A^+$); 2) PDD non-apathetic patients (PDD-$A^-$); 3) apathetic AD patients (AD-$A^+$); 4) non-apathetic AD patients (AD-$A^-$).

As shown in Table 3, the four subgroups did not differ significantly in relation to age, education, mean MMSE score, mean FAB score and mean CDT score. The performances of the four subgroups were significantly different on verbal immediate and delayed recall, TMT:B, TMT:B-A, and both measures derived from IML test. Post hoc comparisons (Mann-Whitney $U$ test) revealed that PDD-A+ group performed significantly worse than PDD-A- on TMT:B ($U$ test $= 28, p = 0.034$) and TMT:B-A ($U$ test $= 21.5, p = 0.008$). The AD-A+ group made significantly more non reversal responses on IML test than the AD-A-group ($U$ test $= 64$, $p = 0.016$). Last, post-hoc comparisons showed that PDD-A+ patients achieved significantly higher scores than AD-A+ group on both immediate and delayed recall of Rey's 15 words ($U$ test $= 31.5$, $p = 0.001$ and $U$ test $= 13.5$, $p < 0.001$, respectively).

PDD-A+ and PDD-A- groups did not differ for disease duration (Mann-Whitney $U$ test $= 81, p = 0.880$) and severity of motor symptoms, as assessed by means of UPDRS-motor section (Mann-Whitney $U$ test $= 55$, $p = 0.139$); moreover, the two groups did not differ for LEDD (Mann-Whitney $U$ test $= 53.5, p = 0.113$).

As regards behavioral results, significant differences among the four groups were found on IADL and on several subscales of NPI: depression, apathy, anxiety, irritability and disinhibition subscale.

Mann-Whitney $U$ test showed that PDD-A+ had lower functional autonomy and higher score on apathy sub-

Table 3
Cognitive comparisons among apathetic patients with PD-associated Dementia (PDD-A+), non apathetic patients with PD-associated Dementia (PDD-A-), apathetic patients with Alzheimer's disease (AD-A+) and non apathetic patients with Alzheimer's disease (AD-A)

| Neuropsychological parameters | PDD-A+ ($n = 14$) | PDD-A- ($n = 15$) | AD-A+ ($n = 15$) | AD-A- ($n = 17$) | $H$ test | P |
|---|---|---|---|---|---|---|
| **a) Frontal Functions** | | | | | | |
| Phonological fluency | 12.1 ± 9.5 | 17.6 ± 8.8 | 12.5 ± 7.3 | 13.2 ± 9.7 | 4.090 | 0.252 |
| Semantic fluency task | 7.9 ± 3.1 | 10.3 ± 3.9 | 8.3 ± 2.9 | 11.3 ± 9.1 | 4.445 | 0.217 |
| RCPM | 13.6 ± 6 | 15.5 ± 4.7 | 12.3 ± 4.7 | 17.5 ± 6.4 | 7.412 | 0.060 |
| IML: correct responses | 9.1 ± 7.4 | 15.8 ± 9 | 12.7 ± 6.8 | 17.6 ± 6.1 | 10.775 | 0.013 |
| IML: non reversal response | 15.6 ± 8 | 8.1 ± 9.1 | 11.2 ± 6.7 | 5.3 ± 5.4 | 14.172 | 0.003[b] |
| ROCF-copy task | 10.5 ± 8.1 | 15.3 ± 11.1 | 12.7 ± 8.7 | 15.8 ± 11.6 | 2.009 | 0.571 |
| TMT: B | 359.4 ± 142.9 | 234.8 ± 84.3 | 481.7 ± 93.7 | 410.6 ± 129.4 | 19.526 | < 0.001[a] |
| TMT: B-A | 132.1 ± 216.2 | 95.5 ± 73.9 | 318.5 ± 97.7 | 259.6 ± 128.5 | 23.737 | < 0.001[a] |
| Stroop test-interference task | 4.3 ± 5.5 | 3.9 ± 4.7 | 2.8 ± 2.7 | 4.9 ± 3.9 | 2.187 | 0.535 |
| **b) Visuospatial abilities** | | | | | | |
| TMT: A | 175.4 ± 149.2 | 161.4 ± 174.3 | 163.2 ± 88.1 | 134.7 ± 79.8 | 2.516 | 0.472 |
| Apraxia constructional task | 5 ± 2.9 | 6.7 ± 3.1 | 7.2 ± 3.7 | 7.7 ± 4 | 5.642 | 0.130 |
| **c) Memory** | | | | | | |
| **– Long term memory** | | | | | | |
| Immediate Recall | 24 ± 7.4 | 23.2 ± 8.8 | 14.4 ± 3.8 | 17.5 ± 8.1 | 13.475 | 0.004[c] |
| Delayed recall | 4.8 ± 2.5 | 4.1 ± 2.4 | 0.8 ± 0.9 | 2 ± 2.4 | 23.045 | < 0.001[c] |
| ROCF-delayed recall | 2.1 ± 2.8 | 3.9 ± 3.3 | 1.6 ± 3 | 3.8 ± 5.9 | 5.936 | 0.115 |
| **– Short term memory** | | | | | | |
| Verbal span for bysillabic words | 3 ± 1.2 | 3.4 ± 0.8 | 3.1 ± 0.6 | 3.1 ± 0.8 | 2.138 | 0.544 |
| Corsi's block-tapping test | 3.1 ± 1.5 | 3.9 ± 1.4 | 3.8 ± 1.3 | 3.4 ± 1.3 | 4.692 | 0.196 |

RCPM, Raven's Colored Progressive Matrices; IML, Inverse Motor Learning test; ROCF, Rey-Osterrieth complex figure test; TMT, Trail Making Test; [a] = significant difference between PDD-A+ and PDD-A-; [b] = significant difference between AD-A+ and AD-A-; [c] = significant difference between PDD-A+ and AD-A+.

scale than PDD-A- (for IADL: $U$ test $= 54.5$, $p = 0.026$; apathy subscale: $U = 51.5$, $p = 0.018$); AD-A+ had less functional autonomy and higher score on depression subscale than AD-A- (for IADL: $U$ test $= 64.5$, $p = 0.016$; depression subscale: $U$ test $= 58.5$, $p = 0.015$). No significant difference was found between PDD-A+ and AD-A+.

There was a significant difference on CJ-AS among the four groups ($p < 0.001$); in particular, PDD-A+ were assigned significantly higher scores than both PDD-A- and AD-A+ groups (PDD-A+ vs. PDD-A-: $U$ test $= 20$, $p < 0.001$; PDD-A+ vs. AD-A+: $U$ test $= 27$, $p < 0.001$); moreover, AD-A+ scored significantly higher than AD-A- ($U$ test $= 43$, $p = 0.001$).

## 4. Discussion

To the best of our knowledge, this is the first study to compare cognitive correlates of apathy in non-depressed patients with PDD and AD. The exclusion of clinically significant depression allowed us to investigate apathy as an independent behavioral disturbance and not as a core symptom of depression, as it has been often considered [52].

It is worth mentioning that diagnosis of "pure apathy" in our sample was made when scores on both direct and indirect standardized measures converged in identifying relevant apathetic symptomatology (self and informant-report questionnaire, i.e. S-AES and I-AES). By these means we detected "pure apathy" in 14 PDD patients (48% of non-depressed PDD sample) and in 15 AD patients (47% of non-depressed AD patients). These figures are similar to those reported in recent literature [14,53,55].

The existence of a relevant subgroup of patients affected by either AD or PDD showing "pure apathy" confirmed that apathy may occur independently of depression both in AD and PDD, and that this symptomatology may constitute a behavioral disturbance to be specifically searched for. This conclusion is further supported by the finding of significantly reduced autonomy in instrumental activities of daily living in our sample of apathetic PDD and AD patients.

Clinical diagnosis of apathy based on a standardized scale was supported by examiner's clinical judgment, as assessed by the short inventory we proposed here (CJ-AS). However, the CJ-AS revealed more severe phenomenological expression of apathy in PDD patients than in AD patients, suggesting that in our sample apathy manifested itself as a reduction of behavior more strongly in PDD patients than in AD patients. The use of a specific inventory to take into account examiner's observation and to compare data from multiple sources (patients, caregivers and clinical experience) might be useful to implement accurate diagnostic tools for apathy.

In the present study, apathy in the whole sample of patients was associated with reduced semantic fluency, reduced ability to inhibit inappropriate motor responses and lower abstract thinking abilities with respect to non-apathetic patients with neurodegenerative dementias. These results suggested that, independently from clinical diagnosis, apathetic patients showed relevant executive impairments, in line with most previous studies [14,15,20,21,23,25,26]. In other terms, the present data indicated a strong association between apathy and alterations of cognitive functions mediated by prefrontal cortex, since apathetic and non-apathetic patients were matched for age, educational level and severity of general cognitive decline.

The same holds true if data relative to the two degenerative diseases are considered separately. In particular, PDD patients with apathy had reduced working memory and task-switching ability compared to PDD patients without apathy; the AD patients with apathy displayed more frequent imitative response tendencies [55–57] than AD patients without apathy, in analogy with prefrontal patients showing the so-called 'imitation behaviour' (i.e. the tendency to overtly imitate the experimenter) [58]. Finally, the comparison between apathetic PD patients and apathetic AD patients revealed significant differences only in verbal long-term tasks, in which AD patients had poorer performance than PD patients, and not in tasks assessing frontal functions. Taken together, these results could suggest that apathy is associated with a dysexecutive syndrome and may be considered as a behavioral disorder highly suggestive of a Behavioral and Cognitive Dysexecutive Syndrome, as recently proposed by Godefroy and colleagues [59].

In recent years, apathy has been often associated with deficits of the prefrontal-Basal Ganglia circuits, as it can occur after focal lesions of specific structures of the basal ganglia, such as the caudate nuclei and the internal pallidum [60–62], and in degenerative diseases affecting basal ganglia [7–11]. However, only one neuroimaging study in depressed PD patients [63] has directly supported this hypothesis by showing a significant association between apathy and reduced activity in the ventral striatum (see for a review, Benoit and Filippi [64]). More recent PET and resting fMRI studies in PD revealed an association between high apathy scores and abnormalities in several prefrontal regions (including orbitofrontal cortex, cingulated region, dorsolateral cortex, right middle frontal cortex), besides other cortical areas, such as bilateral inferior parietal gyrus, bilateral insula and right precuneus [65–67]. Also in AD, apathy severity has been associated with abnormalities in several cortical regions such as the anterior cingulate gyrus [68] and the left medial frontal cortex [69]. Taken together, these results indicated that apathy in neurodegenerative diseases seems to arise from altered functioning of several cortical areas, mainly of prefrontal areas. Our findings about significant association between apathy and more severe alteration of executive functions found in both PDD and AD patients, indirectly supported the idea that abnormalities in prefrontal cortex play a key role in development of apathetic syndrome, independently from the type of neurodegenerative disease.

In conclusion, we explored the neuropsychological correlates of apathy in non-depressed patients with PDD and AD. We found that apathetic patients with both forms of dementia showed a common neuropsychological and behavioral picture, characterized by defects on selected frontal tasks, thus supporting the existence of an 'apathetic syndrome', characterized by specific cognitive and psychological symptoms.
